# Multimodality imaging: Bird’s eye view from the European Society of Cardiology Congress 2019 Paris, August 31st–September 4th, 2019

**DOI:** 10.1007/s12350-019-01958-8

**Published:** 2019-11-18

**Authors:** Victoria Delgado, Antti Saraste, Marc Dweck, Chiara Bucciarelli-Ducci, Jeroen J. Bax

**Affiliations:** 1 Heart Lung Centrum, Leiden University Medical Center, Leiden, The Netherlands; 2 Turku PET Centre, Turku University Hospital and University of Turku, Turku, Finland; 3 Heart Center, Turku University Hospital, Turku, Finland; 4 BHF Centre for Cardiovascular Sciences, University of Edinburgh, Edinburgh, UK; 5 Bristol Heart Institute, National Institute of Health Research (NIHR) Biomedical Research Centre, University Hospitals Bristol and University of Bristol, Bristol, UK

**Keywords:** Echo, CT, MRI, PET, SPECT

## Abstract

At the European Society of Cardiology (ESC) congress of this year 2019, held in Paris from August 31st to September 4th, 4509 abstracts were presented. Of those, 414 (9%) belonged to an imaging category. Experts in echocardiography (VD), nuclear imaging (AS), cardiac computed tomography (CT) (MD) and cardiovascular magnetic resonance (CMR) (CBD), have selected the abstracts in their areas of expertise that were of most interest to them and are summarized in this bird’s eye view from this ESC meeting. These abstracts were integrated by one of the Editors of the Journal (JB).

## Echocardiography

The new guidelines on chronic coronary syndromes,^1^ dyslipidemia^2^ and diabetes mellitus^3^ include recommendations on the use of imaging for diagnosis and risk stratification of atherosclerotic cardiovascular disease. Detection of carotid atherosclerotic plaque with carotid ultrasound is a modifier of the risk of atherosclerotic cardiovascular disease. In 415 women (62 ± 10 years old, 28% with diabetes mellitus) undergoing stress echocardiography, Gurunathan et al^4^ evaluated the incremental value of carotid ultrasound to detect significant coronary artery disease (CAD). Inducible wall motion abnormalities were detected in 47 (11%) women and 41% presented carotid plaque while carotid intima media thickness > 75th percentile was measured in 15%. Stress echocardiography had a positive predictive value of 51% for detecting > 70% coronary artery stenosis on invasive coronary angiography and improved to 71% when carotid plaque was present. These results have important clinical implications since it is well known that the unique pathophysiological mechanisms causing ischemia and the lower prevalence of significant CAD in women limit the predictive value of conventional stress testing techniques.

Mitral annulus disjunction, defined as a variation in the attachment of the posterior mitral leaflet characterized by a wide separation between left atrial wall and the left ventricular (LV) free wall, is frequently observed in patients with mitral valve prolapse. In a large series of patients with moderate and severe mitral regurgitation due to mitral valve prolapse, Mantegazza et al^5^ described a frequency of mitral annulus disjunction of 21% among 630 patients with Barlow’s disease, which is lower than previously reported. In addition, the series included 349 patients with fibroelastic deficiency in whom the presence of mitral annulus disjunction was observed in 8%. Patients with mitral annulus disjunction were usually younger, had more dilated mitral annulus and more frequently presented bileaflet prolapse as compared to patients without.

Assessment of LV systolic function after ST-segment elevation myocardial infarction (STEMI) has important clinical implications. Speckle tracking echocardiography has provided various parameters that have incremental prognostic value over conventional LV ejection fraction. In 372 patients with STEMI treated with primary percutaneous coronary intervention, Brainin et al^6^ measured early systolic lengthening from speckle tracking derived longitudinal strain. The early systolic lengthening is an index that reflects myocardial viability and is calculated as (100 × [peak positive systolic strain/peak negative global strain]) and averaged for 18 LV myocardial segments. In the segments supplied by the infarct-related artery, the early systolic lengthening was significantly higher than in the remote segments (6.7 ± 6.2% vs 5.0 ± 4.1%, *P* < .001). During a median follow-up of 5 years, 39% of patients reached the composite primary end-point of incident heart failure, myocardial infarction or all-cause mortality. Each 1% increase in early systolic lengthening was associated with 27% increased risk of the composite primary end-point.

**Figure 1:**
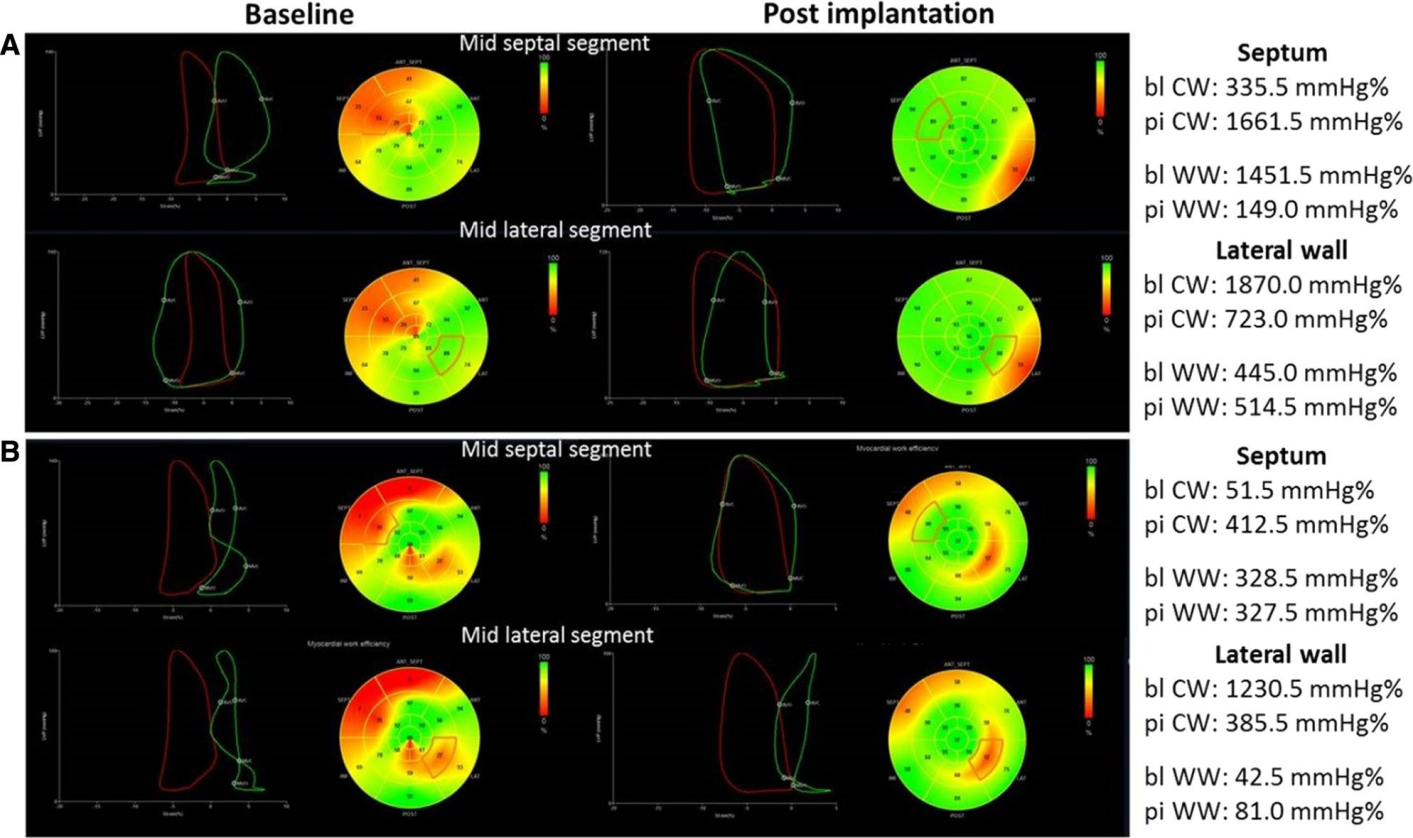
Changes in regional left ventricular myocardial work in responder (**A**) and non-responder (**B**) to cardiac resynchronization therapy. The pressure-strain loops are shown for the global left ventricular (LV) cardiac constructive work (CW) (in red) and for the indicated segments (in green). In the responder patient (**A**), after implantation of cardiac resynchronization therapy there is an increase in the LV CW and this is achieved by significant improvement in CW of the mid septal segment and normalization of the CW of the mid lateral segment. In the non-responder patient (**B**), the CW of the mid septal segment improves but the lateral wall remains impaired resulting in reduced LV CW. Reproduced with permission from Kostyukevich et al^8^*bl*, baseline; *pi*, post-implantation; *WW*, wasted work

Other measures of LV myocardial performance that have been presented at this ESC congress were cardiac power output-to-mass ratio and speckle tracking derived myocardial work indices. In 24,783 patients with preserved LV ejection fraction (≥ 50%) undergoing exercise echocardiography, Anand et al^7^ evaluated the prognostic relevance of peak exercise cardiac power output-to-mass ratio and power reverse (increase in cardiac power output-to-mass ratio from rest to peak exercise). Patients with significant valvular heart disease or right ventricular systolic dysfunction were excluded. Cardiac power output-to-mass ratio is calculated as (0.222 × cardiac output x mean blood pressure/LV mass) and expressed in Watts/100 g myocardium. Lower peak exercise cardiac power output-to-mass ratio and power reserve were independently associated with increased risk of all-cause mortality. Patients within the lowest power reserve quartile (< 0.95 W/100 g) but with otherwise normal stress test had similar survival as those with ischemia on stress test or ischemic cardiomyopathy. In patients with heart failure treated with cardiac resynchronization therapy, regional measures of myocardial work based on speckle tracking echocardiography provide further insights into the response to the device therapy. Regional LV myocardial work is calculated by integrating non-invasive blood pressure measurements, timing of mitral and aortic valve opening and closure and speckle tracking derived LV longitudinal strain. The pressure-strain loops are then derived and the constructive work and wasted work are calculated. Kostyukevich et al^8^ demonstrated that immediately after cardiac resynchronization therapy implantation, responders (patients who showed at 6 months follow-up LV reverse remodeling) showed improvement in constructive work (433.0 [254.5;686.5] mmHg% to 664.5 [424.5;977.8] mmHg%; *P* < .001) and wasted work (305.0 [169.0;461.3] mmHg% to 145.0 [80.0;306.3] mmHg%; *P* = .005) of the septal wall whereas the lateral wall showed normalization of the constructive work with slight increase in wasted work (Figure 1). In contrast, non-responders only showed a decrease in the wasted work of the septal wall.

Based on 3-dimensional echocardiography, the geometry and function of the right ventricle can be fully appreciated. Kovacs et al^9^ described the components of the right ventricular function in 231 healthy individuals: the longitudinal and radial ejection fraction contribute equally to the global right ventricular ejection fraction whereas the anteroposterior ejection fraction has less contribution. Interestingly, the anteroposterior ejection fraction showed a decline with age. The relative contributions of each ejection fraction to the global right ventricular ejection fraction in pathological situations needs to be elucidated in future studies.

## Nuclear Imaging

Machine learning may be helpful in interpretation of imaging data through complex data-pattern recognition. The study of Hu et al^10^ aimed to develop a machine learning computer score derived from stress imaging and clinical data, which indicates if the rest scan could be automatically canceled during myocardial perfusion imaging. The study population consisted of 20,414 patients from the REFINE SPECT registry who had undergone rest/stress single-photon emission computerized tomography (SPECT). There were 3542 adverse events including revascularization, death, myocardial infarction or unstable angina during 4.7 years of follow-up. Machine learning could be used to automatically cancel the rest myocardial perfusion scan with the same proportion (60%) as using normal visual reading, but with significantly lower rate of adverse events after stress-only scan (annual rate 1.5% vs 2.1%) indicating that the rest scan could be safely canceled based on machine learning. Juarez-Orozco et al^11^ evaluated the feasibility and performance of deep learning survival analysis in predicting outcomes in a registry of 951 patients with suspected CAD who underwent cardiac hybrid positron emission tomography/computed tomography (PET/CT). Cox-Nnet (a deep survival neural network) evaluation of PET perfusion and CT angiography data outperformed categorical expert interpretation and coronary calcium score (c-index = 0.75, 0.54 and 0.65, respectively) in predicting the occurrence of myocardial infarction or death during follow-up. The authors conclude that deep learning survival analysis is feasible in the evaluation of cardiovascular prognostic data and may facilitate prediction of outcomes.

**Figure 2:**
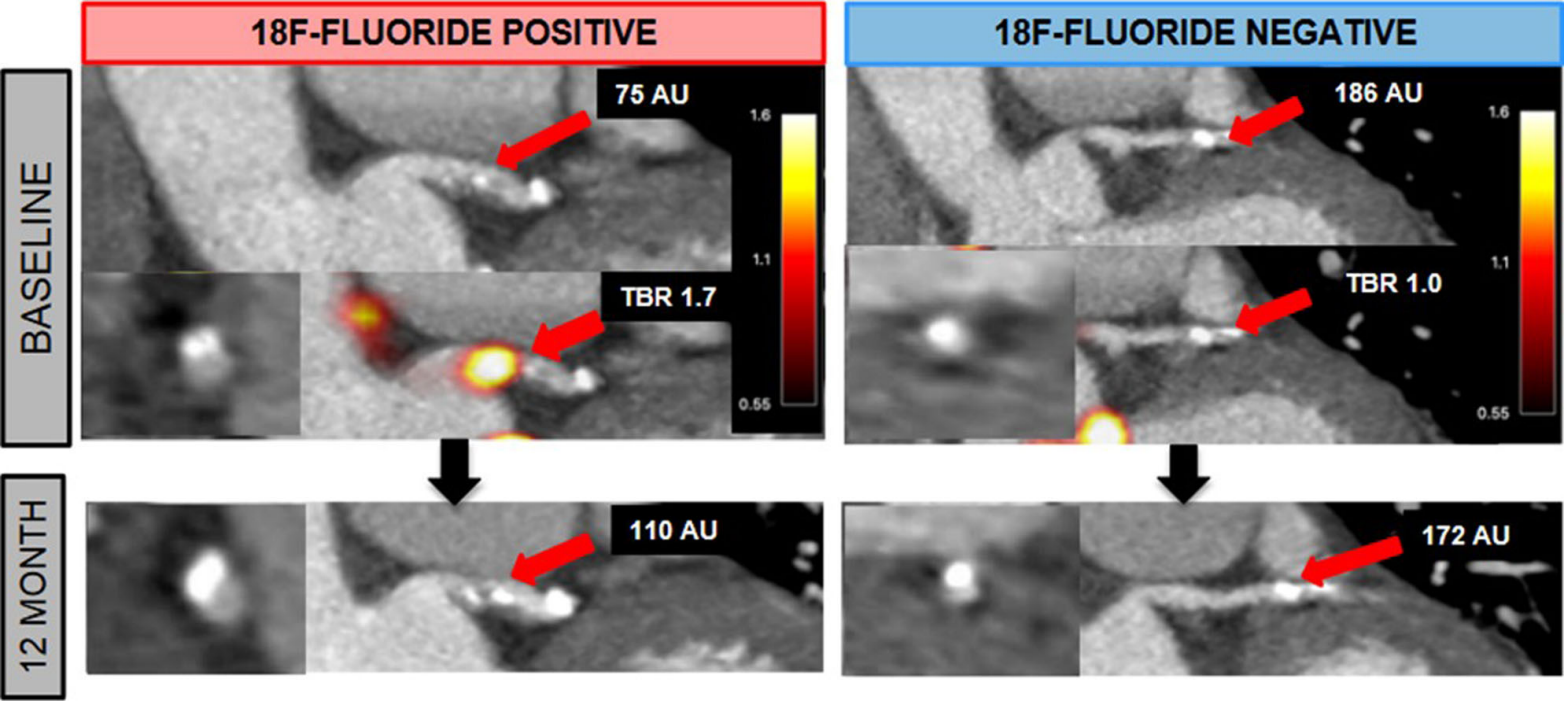
^18^F-sodium fluoride positron emission tomography to predict progression of coronary calcification. Examples of two patients undergoing ^18^F-sodium fluoride (^18^F-NaF) positron emission tomography (PET) and coronary computed tomography angiography at baseline and follow-up. The patient showing uptake of ^18^F-NaF (18F-fluoride positive) had progression of coronary calcification at follow-up whereas the patient without uptake (18F-fluoride negative) did not show changes. Reproduced with permission from Doris et al^12^

^18^F-sodium fluoride (^18^F-NaF) PET provides an opportunity to assess microcalcification in the coronary arteries. Doris et al^12^ evaluated the relationship between coronary ^18^F-NaF uptake and progression of coronary calcification in 185 patients with multivessel CAD (Figure 2). Patients with coronary ^18^F-NaF uptake at baseline (*n* = 116) demonstrated more rapid progression of coronary calcification during 1 year of follow-up than patients without ^18^F-NaF uptake [change in Agatston score 97 (39-166) vs 35 (7-93), *P* < .001]. The authors conclude that ^18^F-NaF PET not only identifies patients with more advanced coronary calcification, but also patients with rapid progression of coronary calcification during 1-year.

The value of hybrid ^18^F-fluorodeoxyglucose (^18^F-FDG) PET and CMR in predicting recovery of wall motion after percutaneous coronary intervention of chronic total coronary occlusion was studied by Vitadello et al.^13^ In 49 patients with chronic total coronary occlusion and wall motion abnormality in the corresponding coronary territory, almost one third of the dysfunctional segments showed discordant imaging findings: 4% were non-viable by ^18^F-FDG-PET, but viable by CMR, whereas 26% were viable by ^18^F-FDG PET, but non-viable by CMR. The combination of PET and MR showed better accuracy in predicting improvement of regional wall motion after revascularization of chronic total coronary occlusion than either ^18^F-FDG PET or CMR alone (area under ROC curve 0.72, 0.58 and 0.66, *P* = .002). These results suggest that hybrid PET/CMR may identify small amounts of viable myocardium in scarred segments that are capable of improving function after revascularization. In addition to viable myocardium, ^18^F-FDG-PET can be used to detect active cardiac inflammation after suppression of physiological myocardial glucose uptake. The study by Protonotarios et al^14^ evaluated ^18^F-FDG PET in 18 patients with an arrhythmogenic cardiomyopathy diagnosed according to the 2010 Task Force criteria. ^18^F-FDG uptake was common in patients with desmosomal gene positive arrhythmogenic cardiomyopathy and clinically active disease. All cases with ^18^F-FDG uptake showed gadolinium late-enhancement, but they were spatially discordant suggesting that ^18^F-FDG PET and CMR provide complementary information on tissue characterization also in arrhythmogenic cardiomyopathy.

^18^F-FDG PET has been shown to be useful for the diagnosis of infective endocarditis, but its prognostic value is unknown. Sovannarith et al^15^ evaluated the prognostic value of ^18^F-FDG PET/CT in patients with definitive prosthetic valve endocarditis (*n* = 109) or native valve endocarditis (*n* = 64) during one-year of follow-up. Valvular uptake of ^18^F-FDG was present in 82% of patients with prosthetic valve endocarditis and it was associated with higher rate of a composite end-point of death, recurrence of infective endocarditis, acute cardiac failure, cardiovascular hospitalization, and new embolic event (odds ratio = 2.6, 95% confidence interval 1.04-6.6, *P* = .04). In native valve endocarditis, valvular ^18^F-FDG uptake was not associated with the composite end-point. However, ^18^F-FDG uptake graded as moderate or high vs. absent or mild was predictive of embolic events in both prosthetic and native valve endocarditis. Therefore, ^18^F-FDG PET/CT is predictive of major cardiac events in prosthetic valve endocarditis and embolic events in both prosthetic and native valve endocarditis during the first year following infective endocarditis.

## Computed Tomography

Computed tomography coronary angiography (CTCA) gained a class I recommendation for the assessment of stable patients with chest pain in the 2019 ESC guidelines launched in Paris.^1^ Concerns have been expressed that the widespread use of CTCA would lead to an increased rate of invasive coronary angiography. Weir-McCall et al^16^ examined trends in the use of imaging for the investigation of CAD in England before and after the 2016 National Institute for Clinical Excellence (NICE) guidelines. These recommended CTCA as the first line imaging test in patients with chest pain. Importantly the NICE guidelines resulted in a fall, not an increase in invasive coronary angiography despite increased rates of CTCA. In addition, centers with the greatest growth in CTCA had the greatest fall in SPECT utilization so that overall imaging costs were unchanged.

There was a clear focus at ESC 2019 on CT studies examining coronary plaque and pericoronary fat. Deseive et al^17^ measured the total coronary plaque volume (both calcific and non-calcific) on CTCA in 1577 patients and then followed them up for 10.4 years for the primary end-point of cardiac death and acute coronary syndrome. The primary end-point was met in 59 patients. Total plaque volume provided incremental prognostic information to a model incorporating cardiovascular risk factors and the severity of obstructive CAD, whereas CT calcium scoring did not. This study highlights the important prognostic role that measures of total coronary plaque volume on contrast CT angiography might play in clinical practice and highlights their potential advantages compared to CT calcium scoring. In a hybrid PET and CT study, Kwiencinski et al^18^ investigated the relationship of low-density plaque on CT with uptake of the PET tracer ^18^F-NaF. Detailed analysis of the coronary plaque composition and ^18^F-NaF activity was performed in 20 patients undergoing hybrid PET/CT coronary angiography. They demonstrated that coronary vessels with increased ^18^F-NaF activity had higher non-calcific and low-density plaque volumes. Moreover, low-density plaque volume was an independent predictor of ^18^F-NaF activity. This study supports the hypothesis that patients with low-density plaque have increased coronary plaque activity, which might explain the increased event rate observed in these patients.

Commandeur et al^19^ used machine learning to perform fully automated quantification of the epicardial adipose tissue in 850 non-contrast calcium scoring CT scans. Automated quantification took less than 2 seconds and demonstrated close agreement to epicardial adipose volumes measured manually by experts who took 15 minutes. The same investigational group also used a machine learning approach to determine whether CT calcium scores and epicardial fat volumes (quantified automatically using the above method) alongside clinical information might improve risk prediction in 2071 patients from the EISNER trial.^20^ After long-term follow-up they demonstrated that a machine learning approach integrating clinical and quantitative imaging-based variables improved the prediction of myocardial infarction and cardiac death in patients undergoing CT calcium scoring compared to standard risk assessment methods.

**Figure 3:**
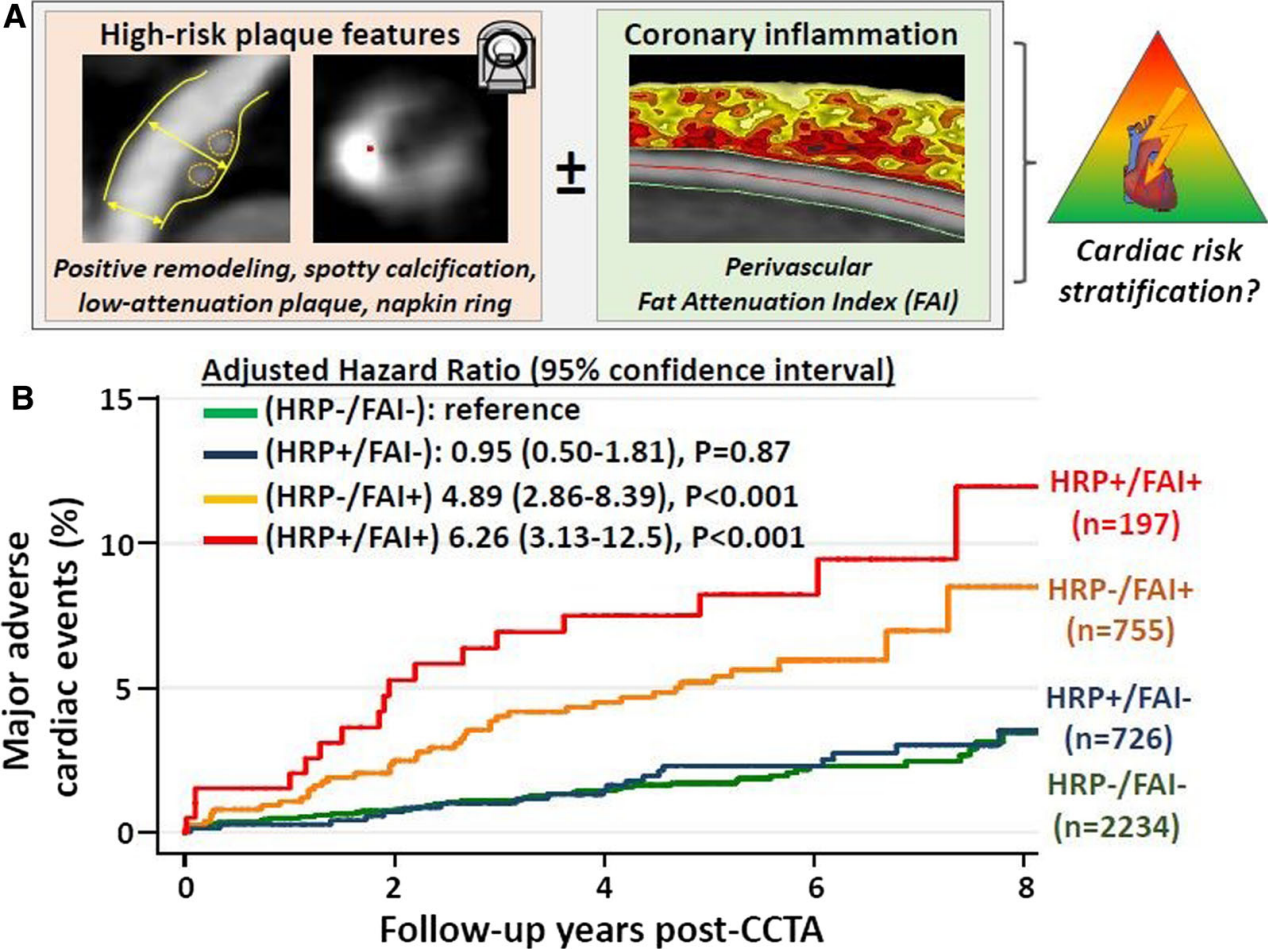
Assessment of high-risk plaque features and coronary inflammation on computed tomography coronary angiography and prediction of major adverse cardiac events. (**A**) Shows the high-risk plaque features (HRP) combined with the assessment of perivascular fat attenuation index (FAI). (**B**) shows the Kaplan Meier curves for occurrence of major adverse cardiovascular events; patients with HRP features and coronary inflammation based on FAI had significantly worse prognosis as compared to the other groups of patients. Reproduced with permission from Oikonomou et al^21^

Finally, Oikonomou et al^21^ investigated the composition of pericoronary fat on contrast CTCA as a marker of coronary plaque inflammation and assessed its prognostic value alongside high-risk coronary plaque features assessed on the same scan. The rationale for this approach is that atherosclerotic inflammation alters the structure of the pericoronary fat by reducing its fat content: an effect that can be captured using the perivascular Fat Attenuation Index (FAI) (Figure 3, panel A). The authors investigated 3912 patients from the CRISP-CT study that had undergone clinically indicated CTCA. High-risk plaque (HRP) was defined as the presence of 1 or more of the following: positive remodeling, low-attenuation plaque, spotty calcification or napkin ring sign. After 5.6 years of follow-up, there were 91 confirmed major adverse cardiovascular events (cardiac mortality or non-fatal myocardial infarction) (Figure 3, panel B). Patients with both HRP and high FAI had a 6.3-fold higher adjusted risk of major adverse cardiovascular events compared to individuals with neither feature. Patients without HRP but a high FAI had a 4.9-fold higher adjusted risk of major adverse cardiovascular events. However, in patients with low FAI, there was no significant difference in the prospective risk of major adverse cardiovascular events between patients with and without HRP. These data indicate that CTCA-derived FAI can improve patient risk stratification, supplementing anatomical plaque assessments with a functional marker of disease activity.

## Cardiovascular Magnetic Resonance

Non-invasive functional imaging for myocardial ischemia, such as stress CMR, to diagnose CAD remains as class IB recommendation in current ESC guidelines. In this ESC congress, several abstracts provided additional information on the use of this imaging modality in patients with suspected or known CAD. Haberkorn et al^22^ performed a rigorous systematic review and meta-analysis to compare the diagnostic accuracy of vasodilator stress perfusion CMR and dobutamine stress echocardiography using coronary angiography (coronary stenosis > 70%) or fractional flow reserve (< 0.80) as a reference standard for obstructive CAD. Of 5634 studies, 47 prospective studies were selected, totalling 4742 patients. The sensitivity, specificity and diagnostic odds ratio for vasodilator stress CMR were 0.88 (95% CI) 0.850.90), 0.84 (95% CI 0.810.87), and 38 (95% CI 2949), and for dobutamine stress echocardiography 0.72 (95% CI 0.610.81), 0.89 (95% CI 0.830.93), and 20 (95% CI 946), respectively. Vasodilator stress CMR refined the post-test probability of CAD based on lower positive and negative likelihood ratios as compared to dobutamine stress echocardiography (5.5 and 0.14 vs 6.3 and 0.3, respectively). On behalf of the GadaCAD writing group, Andrew Arai presented the diagnostic accuracy of gadobutrol-enhanced CMR to detect significant CAD combing the results of the GadaCAD1 (*n* = 376 patients) and GadaCAD2 (*n* = 389 patients) phase III clinical trials.^23^ Three blinded, core-lab CMR readers per trial (6 observers in total) and 2 core-lab readers of quantitative coronary analysis participated in the analysis. The results showed that the test had 79% sensitivity, 87% specificity, and area under the curve of 0.82 to detect a 70% coronary artery stenosis on quantitative coronary analysis. When a 50% stenosis threshold was used, the area under the curve decreased to 0.77 and the minimum performance threshold was achieved in 1 out of the 6 readers for sensitivity (the lower limit of the 95% CI should be > 60%) and in all the observers for the specificity (the lower limit of the 95% CI should be > 55%). Therefore, these results support the 70% coronary stenosis threshold to define hemodynamically significant stenosis.

Furthermore, stress CMR is an important imaging tool for risk stratification of patients with known or suspected CAD. In a prospective multicentre registry including 6389 patients, Marcos Garces et al^24^ evaluated the association between the ischemic burden and all-cause mortality. During a median follow-up of almost 6 years, 717 (11.2%) patients died. The extent of ischemic burden (per 1 segment increase) on vasodilator stress CMR was independently related to long-term all-cause death (HR 1.05 [95% CI 1.031.07], *P* < .001). The interaction between coronary revascularization and vasodilator stress CMR was evaluated in 1034 patients (517 revascularized and 517 non-revascularized strictly 1:1 matched for age, diabetes mellitus, male sex, LVEF, ischemic burden and necrosis extent). In patients with more than 5 ischemic LV segments, revascularization was associated with improved survival (mortality rates for revascularized and non-revascularized patients: 9.3% vs. 16.3%, respectively: HR 0.56 [95% CI 0.320.98], *P* = .02) but independently increased the risk in patients with ≤5 ischemic LV segments (mortality rates for revascularized and non-revascularized patients: 16.2% vs 11.3%, respectively: HR 1.59 [95% CI 1.032.45], *P* = .037).

**Figure 4:**
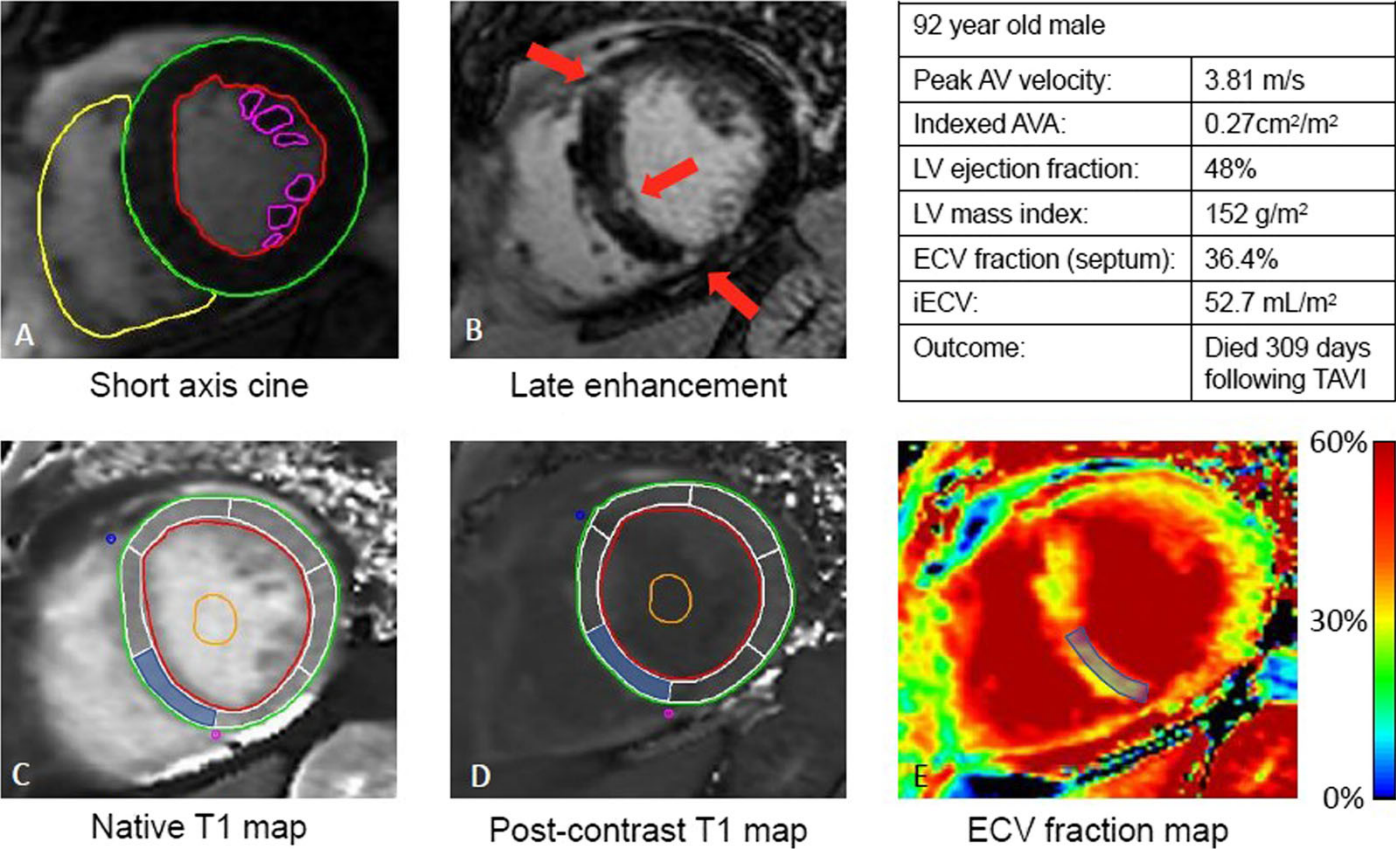
Comprehensive CMR assessment in valvular heart disease: ventricular function (**A**), late gadolinium assessment (**B**), native T1 mapping (**C**), post-contrast T1 mapping (**D**) and extracellular volume of distribution map (**E**). Reproduced with permission from Everett et al^25^

In the field of valvular heart disease, the use of CMR is increasing. Everett et al^25^ investigated the association between the presence and extent of focal and diffuse LV myocardial fibrosis with the disease severity and long-term clinical outcome in 440 patients (70 ± 10 years, 59% male) with severe aortic stenosis scheduled for aortic valve intervention. LV myocardial fibrosis was quantified using CMR imaging with late gadolinium enhanced and T1 mapping techniques (Figure 4). Extracellular volume fraction [ECV%] calculated from T1 mapping images (mean 27.7 ± 3.6%) correlated significantly with lower peak aortic jet velocity, larger LV mass, lower LVEF, and presence of late gadolinium enhancement. Furthermore, ECV independently associated with all-cause mortality following adjustment for age, sex, impaired LV ejection fraction and presence of late gadolinium enhancement (HR per unit increase in ECV: 1.10, 95%, (95% CI 1.021.19), *P* = .013). The authors concluded that diffuse LV myocardial fibrosis (ECV) correlates with LV dysfunction and it is a strong independent predictor of late all-cause mortality.

Romano et al^26^ investigated whether LV GLS measured with CMR feature tracking has prognostic value in individuals with preserved LVEF. Consecutive patients (*n* = 1274) with preserved LVEF (> 50%) and a clinical indication for CMR were included in a multicenter American registry. The median value of LV GLS was − 20%. During a median follow-up of 6.2 years, 115 patients died. Patients with an LV GLS ≥ − 20% had significantly reduced event-free survival compared to those with GLS<− 20% (log-rank *P* < .001). On Cox multivariable regression, each 1% worsening in LVGLS was associated with a 23.6% increased risk of death after adjustment for clinical and imaging risk factors.

Finally, Menacho Medina et al^27^ presented the effort of 4 countries (Argentina, Peru, India and South Africa) and 12 centers to implement an educational program and rapid CMR acquisition protocol (cardiac anatomy, function and tissue characterization) in 510 patients with cardiomyopathy. The mean duration was 21 ± 6 minutes for a contrast study and 12 ± 3 minutes for a non-contrast study. The measured clinical impact of CMR was (a) identification of new diagnosis in 105 patients (21%), (b) change/addition of medication in 128 patients (25%), (c) new procedure (intervention/surgery in 31 patients, 6% and invasive angiography or biopsy in 25 patients, 5%), or (d) hospital discharge/admission in 15 patients (3%). The investigators concluded that CMR can be easily be delivered in the developing countries with existing technology using faster protocols, leading to a significant clinical impact in 60% of patients.

## Abbreviations

CAD: Coronary artery disease
CMR: Cardiac magnetic resonance
CRT: Cardiac resynchronization therapy
CT: Computed tomography
CTCA: Computed tomography coronary angiography
ECV: Extracellular volume
ESC: European Society of Cardiology
FAI: Fat attenuation index
FDG: Fluorodeoxyglucose
HRP: High-risk plaque
LGE: Late gadolinium enhancement
LV: Left ventricular
LVEF: Left ventricular ejection fraction
PET: Positron emission tomography
SPECT: Single-photon emission computerized tomography
STEMI: ST-segment elevation myocardial infarction
